# Olive Leaf Extract Suppresses Sebogenesis and Inflammation via AKT/ERK and SREBP-1/PPAR-γ Signaling in Human Sebocytes

**DOI:** 10.3390/cimb48060549

**Published:** 2026-05-23

**Authors:** Jeeyoung Kim, Ye-Won Jo, Weon Jeong Bang, Kwang Won Lee, Yung Hyup Joo, Sung Hyeon Lee, Chang-Seok Lee

**Affiliations:** 1 Research and Development Center, Durae Corporation, Anyang Trade Center 14F, 161, Simin-daero, Dongan-gu, Anyang 14048, Gyeonggi-do, Republic of Korea; kjy@durae.co.kr (J.K.); bwj2664@durae.co.kr (W.J.B.); eecl308@naver.com (K.W.L.); yhjoo@durae.co.kr (Y.H.J.); harrison@durae.co.kr (S.H.L.); 2 Department of Senior Healthcare, Eulji University, Sujeong-gu, Seongnam 13135, Gyeonggi-do, Republic of Korea; choywalice@naver.com; 3 Cosmetic Science Major, Eulji University, Sujeong-gu, Seongnam 13135, Gyeonggi-do, Republic of Korea

**Keywords:** olive leaf extract, oleuropein, sebum regulation, inflammation, *Cutibacterium acnes*, PPAR-γ, SREBP-1, collagen gel contraction, dermal remodeling

## Abstract

This study evaluated olive leaf extract (OLE) as a multifunctional dermocosmetic candidate for sebum-related and inflammatory responses relevant to oily and acne-prone skin using an axis-aligned in vitro panel: (i) sebocyte lipogenesis, (ii) inflammatory mediator production in keratinocytes, and (iii) fibroblast-mediated collagen gel contraction. In addition, supportive mechanistic evidence for the sebum-related effects of OLE was obtained by examining signaling proteins associated with sebocyte lipogenesis, including PPAR-γ and SREBP-1. As a result, OLE significantly inhibited linoleic acid-induced lipid accumulation in SEB-1 sebocytes without cytotoxicity. In HaCaT keratinocytes, OLE significantly reduced the production of pro-inflammatory cytokines, including IL-8, TNF-α, and PGE_2_, induced by *Cutibacterium acnes* or UVB. In dermal fibroblast-containing collagen gels, OLE enhanced fibroblast-mediated gel contraction. Additionally, analysis of the main mechanisms of lipid inhibition using SEB-1 sebocytes revealed that OLE exerts a dual regulatory role in lipid synthesis and inflammation by downregulating AKT and ERK phosphorylation and inhibiting PPAR-γ and SREBP-1 expression. Furthermore, among the tested extracts, the 70% ethanol extract (OLE70) exhibited the strongest antioxidant activity, the greatest gel contraction response, and the highest content of oleuropein, a major bioactive phenolic compound derived from olive. Like OLE, oleuropein also showed sebum-regulatory activity by reducing lipid accumulation in SEB-1 sebocytes, an inhibitory effect on IL-8 expression in HaCaT keratinocytes, and an inhibitory effect on the expression of PPAR-γ and SREBP-1, which are involved in sebum secretion. Taken together, these findings suggest that OLE and its major phenolic constituent, oleuropein, may modulate sebum-related, inflammatory, oxidative, and dermal remodeling-associated responses in skin cell models. These results should be interpreted as exploratory and provide a basis for further mechanistic and translational investigation.

## 1. Introduction

Sebum, secreted by sebaceous glands, constitutes a major lipid barrier that protects and lubricates the skin surface [1]. However, when its secretion becomes excessive or dysregulated, the accumulated lipids can undergo peroxidation, inducing oxidative stress and inflammatory responses within the pilosebaceous unit [2,3]. This dysregulation of sebaceous function is regarded as a major contributor to oily skin disorders and acne pathogenesis [4].

Excessive sebum production and the subsequent cascade pore occlusion, *Cutibacterium acnes* (*C. acnes*) proliferation, and localized inflammation are fundamental features of acne vulgaris. The pathophysiology of acne involves complex interactions among hyperkeratinization, microbial colonization, sebaceous gland hyperactivity, and inflammation [4,5,6]. Thiboutot et al. further highlighted that acne should be regarded as a chronic inflammatory disease with recurrent relapses and a long disease course, in which four key pathogenic events—excessive sebum production by the sebaceous gland, follicular colonization by *C. acnes*, altered keratinization, and the release of inflammatory mediators including matrix metalloproteinases—cooperate from the very early stages of lesion formation [7]. In addition, *C. acnes* activates innate immunity through pattern recognition receptors including TLR2 and TLR4, inducing production of pro-inflammatory cytokines such as IL-1α/β, IL-6, IL-8, and TNF-α in keratinocytes and sebocytes, thereby amplifying local inflammatory responses in acne pathogenesis [8,9,10]. Thus, sebocytes are now recognized to exert immunomodulatory functions, maintaining skin barrier homeostasis beyond lipid synthesis [2,11,12].

Sebaceous lipid metabolism is primarily controlled by key transcription factors such as sterol regulatory element binding protein 1 (SREBP-1) and peroxisome proliferator-activated receptor γ (PPAR-γ). SREBP-1 activates genes related to fatty acid and cholesterol biosynthesis, while PPAR-γ contributes to lipid storage, sebocyte differentiation, and anti-inflammatory regulation [13,14]. Recent studies demonstrated that pharmacological modulation of PPAR-γ or SREBP-1 alters sebaceous lipid homeostasis and inflammatory balance, suggesting these pathways as viable therapeutic targets for acne [13,14]. PPAR-γ modulates sebaceous lipogenesis and exerts anti-inflammatory effects by antagonizing transcription factors including NF-κB and AP-1, thereby preventing nuclear translocation of inflammatory mediators [2].

Natural compounds targeting these signaling pathways have attracted increasing attention as potential alternatives to synthetic agents. Among them, olive leaf (*Olea europaea* L.) extract (OLE) has emerged as a promising dermatological and cosmetic ingredient due to its rich phenolic profile, particularly oleuropein. Oleuropein, a secoiridoid glycoside unique to olive trees, exhibits potent antioxidant [15], anti-inflammatory, and photoprotective activities [16]. Barbaro et al. demonstrated that oleuropein markedly suppresses pro-inflammatory mediators such as TNF-α, IL-1β, IL-6, iNOS, COX-2, and NO in various in vivo models, indicating a broad anti-inflammatory potential that is relevant to acne pathogenesis, in which these cytokines and mediators aggravate sebaceous inflammation [17]. These bioactivities enable olive-derived polyphenols to attenuate oxidative stress and UV-induced inflammation, thereby contributing to the maintenance of skin homeostasis [16].

In addition, several studies have demonstrated that olive polyphenols regulate extracellular matrix (ECM) remodeling and lipid metabolism in skin cells [18], supporting their potential as multifunctional agents for skin protection, soothing, and anti-aging formulations. Consistent with these observations, an excisional wound model revealed that topical application of an Arbequina olive leaf extract (2% *w*/*v*) significantly accelerated wound closure compared with vehicle, increased TGF-β expression in wound tissue, and markedly reduced pro-inflammatory TNF-α and IL-1β levels; in parallel, lignan-rich constituents of the extract potently inhibited MMP-1 activity in vitro (IC_50_ ≈ 88 nM), further supporting a direct role of olive leaf-derived components in ECM remodeling and the resolution of cutaneous inflammation [19]. In line with these findings, oleuropein was recently reported to modulate oxidative stress and inflammatory responses in human dermal fibroblasts, improving cellular redox balance and supporting extracellular matrix homeostasis, which further corroborates its contribution to dermal firmness and remodeling [20]. Beyond sebocyte lipogenesis and keratinocyte-driven inflammation, acne is also increasingly recognized to involve the structural remodeling of the dermal compartment, particularly in persistent or recurrent lesions that may progress to textural changes and scarring. Inflammatory acne lesions exhibit molecular features linked to collagen degradation and extracellular matrix remodeling in vivo, and histological analyses of atrophic acne scars have demonstrated reduced dermal thickness together with loss of pilosebaceous units. In addition, *C. acnes* has been reported to stimulate TNF-α-dependent proMMP-2 expression in human dermal fibroblasts, further linking acne-associated inflammation to dermal matrix remodeling. In this context, fibroblast collagen gel contraction may be regarded as a wound-healing/remodeling-related functional readout of fibroblast-mediated matrix contractility and post-inflammatory structural support, rather than as a direct model of acne pathogenesis [21,22,23,24]. Also, clinical studies have shown that OLE-containing formulations improve facial skin hydration, reduce sebum levels, and enhance overall skin condition [25]. Additionally, experimental data indicate that OLE and oleuropein inhibit UVB-induced oxidative stress, matrix metalloproteinase (MMP) activation, and COX-2 expression in skin tissues [26]. Despite these promising results, direct evidence demonstrating modulation of lipid metabolism and SREBP-1/PPAR-γ signaling by OLE in sebocytes remains limited.

Therefore, this study was designed to evaluate OLE using a mechanistic-plus-functional framework aligned with key acne-relevant skin axes. We primarily interrogated the sebocyte lipogenesis axis and its upstream signaling (SREBP-1/PPAR-γ and AKT/ERK) to define a mechanistic basis for sebum-related modulation. In parallel, we assessed whether OLE modulates pathogen-relevant inflammatory mediator outputs in keratinocytes under *C. acnes* stimulation and under a standardized oxidative inflammatory stress condition. Finally, we included a fibroblast collagen contraction assay as a dermal remodeling-related functional endpoint relevant to fibroblast-mediated matrix contractility and post-inflammatory structural support, without positioning it as a direct bacterial acne model or as direct evidence of anti-acne efficacy.

## 2. Materials and Methods

### 2.1. Plant Material and Extraction

Finely comminuted olive (*Olea europaea* L.) leaves were used as the botanical starting material. The olive leaves were cultivated in Seogwipo-si, Jeju-do, Republic of Korea, and purchased from OLIVE STANDARD (Jeju, Republic of Korea). Dried leaf powder (100 g) was transferred to a jacketed glass extractor and extracted with a mixture of absolute ethanol (99.9%) and purified water in a 20:1 (*w*/*w*) solvent-to-solid ratio to prepare olive leaf 70% (*w*/*w*) ethanolic extract (OLE70) and olive leaf 50% (*w*/*w*) ethanolic extract (OLE50). For the olive leaf aqueous extract (OLW), purified water alone was employed in the same twenty-fold (*w*/*w*) solvent-to-solid ratio. Extractions were carried out at 60 °C for 3 h with agitation at 300 rpm. Upon completion, the mixtures were allowed to cool gradually to room temperature (≈25 °C) over 3 h. The cooled extracts were clarified by vacuum filtration through 0.5 µm filter paper (Hyundai Micro, Seoul, Republic of Korea), and the filtrates were concentrated under reduced pressure at 65 °C using a rotary evaporator (Eyela, Tokyo, Japan) until the bulk of the solvent was removed. Residual solvent was eliminated by lyophilization at −82 to −78 °C for 24 h. Upon completion of lyophilization, the extract powders obtained under each condition (OLE70, OLE50 and OLW) were stored at −20 °C until required for subsequent experiments.

### 2.2. Determination of Oleuropein by HPLC

Oleuropein reference standard (purity ≥ 98%) was purchased from ChemFaces (Wuhan, Hubei, China). Acetonitrile (≥99.9%, HPLC grade) was purchased from Honeywell. Phosphoric acid (85%, HPLC grade) was purchased from Daejung Chemicals & Metals Co., Ltd. (Siheung, Republic of Korea), and used as a mobile-phase modifier at a final concentration of 0.1% (*v*/*v*) in water. Oleuropein was quantified in the olive leaf extract using a high-performance liquid chromatography (HPLC) system equipped with a photodiode-array detector (PDA 2998, Waters, MA, USA). Separation was performed on a Phenomenex Luna C18 column (5 µm, 250 × 4.6 mm i.d.), which was thermostatted at 30 °C throughout the run. Each sample was diluted with the appropriate solvent, sonicated for 10 min, passed through a 0.45 µm PTFE membrane filter, transferred to an amber glass HPLC vial, and a 10 µL portion was injected into the system via the autosampler for analysis. The mobile phase consisted of solvent A (0.1% *v*/*v* phosphoric acid in water) and solvent B (acetonitrile), delivered at 1.0 mL/min under the gradient scheme detailed in Table 1. Oleuropein (62.5–1000 µg/mL) was monitored at 238 nm with the photodiode-array detector. All measurements were carried out in triplicate to ensure analytical reproducibility. The limit of detection (LOD) and limit of quantification (LOQ) were calculated according to the ICH Q2(R1) guidelines using the equations LOD = 3.3σ/S and LOQ = 10σ/S, where σ is the standard deviation of the y-intercept and S is the slope of the calibration curve. Inter-day repeatability was assessed by performing triplicate analyses of an oleuropein standard solution (250 μg/mL) on three consecutive days.

### 2.3. Cell Culture and Treatment

SEB-1 (the immortalized human sebaceous gland cell line) and HaCaT (the human keratinocytes cell line) were purchased from Koma Biotech (Seoul, Republic of Korea). SEB-1 sebocytes were cultured in DMEM/Ham’s F-12 (3:1) medium (Invitrogen, Carlsbad, CA, USA) containing 5.5 mM glucose, 2.5% fetal bovine serum (FBS; Welgene, Gyeongsan, Republic of Korea), 1.8 × 10^−4^ M adenine, 0.4 µg/mL hydrocortisone, 10 ng/mL insulin, 3 ng/mL epidermal growth factor (EGF), and 1.2 × 10^−10^ M cholera toxin. Except for the medium and FBS, all other supplements were purchased from Sigma-Aldrich (St. Louis, MO, USA) [27]. HaCaT keratinocytes were grown in DMEM (Welgene, Gyeongsan, Republic of Korea) supplemented with 10% FBS and 1% penicillin–streptomycin mixture. All cells were cultured at 37 °C in a humidified environment with 5% CO_2_. Cells were cotreated with OLE or oleuropein and stimulated with linoleic acid (100 μM), *C. acnes* lysate (10%), or UVB (15 mJ/cm^2^) for 24 h.

### 2.4. Cell Viability (WST-1 Assay)

Cell viability was determined using a WST-1 assay kit (EZ-Cytox; DoGenBio, Seoul, Republic of Korea) according to the manufacturer’s instructions. Briefly, cells were seeded in 96-well plates, cultured for 24 h, and then treated with different concentrations of the test samples. The cells were incubated for 24 h, and 100 μL of medium containing 10 μL of water-soluble tetrazolium salt (WST) solution was added to each well; then the plates were incubated for 1 h at 37 °C. The absorbance of each well at 450 nm was measured using an Absorbance Microplate Reader (Multiskan GO; Thermo Scientific, Waltham, MA, USA). Cell viability was determined by the equation:

Cell viability (%) = sample treatment group absorbance/control group absorbance × 100.

### 2.5. Lipid Accumulation Assay (Oil Red O and Nile Red Staining)

#### 2.5.1. Oil Red O Staining

Lipid accumulation was quantified and visualized using Oil Red O staining and Nile Red staining. SEB-1 cells are an established immortalized human sebocyte model that retains key sebaceous characteristics, including lipid synthesis and intracellular lipid accumulation in response to lipogenic stimuli [28,29,30]. Resveratrol was included as a reference lipid-lowering control to confirm the responsiveness of the linoleic acid-induced lipid-accumulation assay. Although previous reports have mainly evaluated resveratrol in other human sebocyte models, such as SZ95 cells, and under experimental conditions that are not identical to those used in the present study, resveratrol has been shown to suppress lipid accumulation and lipogenesis-related signaling in human sebocyte systems [31]. Therefore, in the present SEB-1 assay, resveratrol was used as a practical assay-performance reference for Oil Red O and Nile Red-based lipid accumulation, rather than as a direct mechanistic comparator for OLE.

SEB-1 cells were seeded in 24-well culture plates (1 × 10^4^ cells/well). After overnight incubation, the medium was replaced with conditioned medium containing linoleic acid (Sigma-Aldrich) and OLW, OLE50, OLE70, oleuropein, or resveratrol (20 µM) as a positive control for 72 h. Cells were washed with phosphate-buffered saline (PBS; Welgene, Gyeongsan, Republic of Korea), fixed with 4% formaldehyde (BYLABS, Seoul, Republic of Korea) for 10 min, and stained with 0.3% Oil Red O working solution (prepared from 0.5% Oil Red O stock in isopropanol and filtered) for 15 min in the dark. After rinsing with 60% isopropanol, intracellular lipid was eluted with 99% isopropanol, and absorbance was measured at 500 nm.

#### 2.5.2. Nile Red Staining

SEB-1 cells were seeded in 6-well culture plates (5 × 10^4^ cells/well). After overnight incubation, the medium was replaced with a conditioned medium containing linoleic acid (Sigma, St. Louis, MO, USA) and olive leaf extracts (OLW, OLE50, or OLE70) or resveratrol (20 µM) as a positive control for 72 h. The cells were then washed twice with PBS (Welgene, Gyeongsan, Republic of Korea) and fixed with 4% formaldehyde (BYLABS, Seoul, Republic of Korea) for 10 min at room temperature. Subsequently, cells were stained with 1 µg/mL Nile Red working solution (prepared from a 1 mg/mL stock solution in DMSO) for 15 min in the dark. After washing with PBS, 500 μL of PBS was added to each well to prevent cell dehydration, and the samples were examined under a fluorescence microscope (ECLIPSE Ts2-FL; Nikon, Tokyo, Japan) using a 20× objective lens and excitation/emission filters of 485/565 nm for neutral lipids and 540/620 nm for polar lipids.

### 2.6. Cytokine Measurement

Salicylic acid (SA) was included as an acne-related reference compound in the *C. acnes*-stimulated HaCaT keratinocyte model to confirm the responsiveness of the inflammatory cytokine readout. *C. acnes* stimulation has been reported to induce inflammatory cytokine production in human keratinocytes, including IL-8 and TNF-α [32]. Although SA is widely recognized for its keratolytic activity, it has also been reported to have acne-related and anti-inflammatory relevance in cutaneous experimental contexts [33,34]. Therefore, SA was used here as an endpoint-oriented reference control rather than as a mechanism-matched comparator for OLE. For the UVB-induced inflammatory model, indomethacin (10 µM), a cyclooxygenase inhibitor, was used as a pharmacological reference control for COX-linked inflammatory mediator production, particularly PGE_2_, because UVB exposure is closely associated with PGE_2_-mediated skin inflammatory responses [35,36]. Indomethacin was applied under the same treatment schedule as OLE70 in the UVB model to allow for endpoint-based comparison of UVB-induced TNF-α, IL-8, and PGE_2_ suppression.

### 2.7. Antioxidant Assays

#### 2.7.1. 2,2-Diphenyl-1-Picrylhydrazyl (DPPH) Assay

To investigate the antioxidant scavenging activity, 2,2-Diphenyl-1-picrylhydrazyl (DPPH; Sigma-Aldrich, St. Louis, MO, USA) was employed in this study. A 4 mM DPPH solution was prepared in 99% ethanol and stored in the dark. Ascorbic acid (Samchun Chemical, Seoul, Republic of Korea) was used as positive control, while 99% ethanol served as the negative control. The DPPH solution was diluted in a 1:10 ratio before use. After 10 min incubation, absorbance was measured at 520 nm using a microplate reader.

#### 2.7.2. 2,2′-Azino-Bis(3-Ethylbenzothiazoline-6-Sulfonic Acid) (ABTS•^+^) Assay

A 2,2′-azino-bis(3-ethylbenzothiazoline-6-sulfonic acid) (ABTS•^+^) solution was prepared by mixing 7.4 mM ABTS (Sigma-Aldrich) with 2.6 mM potassium persulfate in PBS (Sigma-Aldrich) and stored in the dark for 16 h. Ascorbic acid was used as positive control, and Dimethyl sulfoxide (DMSO; Sigma-Aldrich) served as the negative control. The ABTS•^+^ solution was diluted 1:45, and 190 μL was dispensed per well. After 15 min incubation in the dark, absorbance was measured at 734 nm.

#### 2.7.3. 2′,7′-Dichlorodihydrofluorescein Diacetate (DCF-DA) Assay

HaCaT cells were seeded in 96-well black culture plates (Greiner Bio-One, Kremsmünster, Austria) at 2.5 × 10^4^ cells/well and stabilized for 24 h. The medium was removed and the sample applied, with ascorbic acid serving as a positive control. Buffers were prepared by diluting 10X HBSS (Welgene) with Deionized Water (DIW) to 1X HBSS. For 2′,7′-dichlorodihydrofluorescein Diacetate (DCF-DA) staining, 50 mM stock was prepared in DMSO and diluted to 50 μM in 1X HBSS before use. After 3 h pretreatment with the sample, cells were incubated with 50 μM DCF-DA for 30 min in the dark, irradiated with Ultraviolet B (UVB) light at 30 mJ/cm^2^, and further incubated for 2 h. Reactive Oxygen Species (ROS) levels were quantified with a microplate reader (excitation, 485 nm; emission, 535 nm). Results are presented as values after UVB relative to before UVB.

### 2.8. Collagen Gel Contraction Assay

The collagen gel contraction assay was performed to assess fibroblast-mediated matrix contraction [27]. Type I collagen (Gibco) was diluted with sterile distilled water, mixed with fibroblasts in Medium 106 (Thermo Fisher Scientific, Waltham, MA, USA), and neutralized with 0.5 N NaOH. The collagen–cell mixture was added to 24-well plates and incubated at 37 °C for 2 h to enable gel polymerization. After detaching the gel edges, fresh medium was added to float the gels, which were then incubated overnight. Samples were treated with test materials, while TGF-β_1_ (R&D Systems, MN, USA) and trypsin–EDTA (Welgene) served as positive and negative controls, respectively. Images were captured at 0 h and 24 h using an Amersham Imager LAS (Cytiva, Marlborough, MA, USA), and the gel area was quantified with ImageJ software (1.54r).

### 2.9. Western Blot Analysis

Cells in culture dishes were directly lysed with RIPA buffer (Thermo Fisher Scientific, Waltham, MA, USA) supplemented with a cocktail of proteinase and phosphatase inhibitors (Thermo Fisher Scientific, Waltham, MA, USA). Cell lysates (30 ug) were resolved by sodium dodecyl sulfate–polyacrylamide gel electrophoresis (SDS-PAGE) on 4–12% gels and transferred to nitrocellulose membranes (Invitrogen, Carlsbad, CA, USA). The blots were washed with 10 mM Tris-HCl [pH 7.6], 150 mM NaCl, and 0.1% Tween-20 (TBST), blocked with 5% skim milk in TBST for 1 h at room temperature and incubated for 12 h at 4 °C with primary antibodies (diluted 1:1000) against phosphorylated p-AKT, total AKT, p-ERK, total ERK, PPAR-γ (Cell Signaling Technology, Danvers, MA, USA) and SREBP-1 (Santa Cruz Biotechnology, Inc., Dallas, TX, USA). The membranes were washed with TBST and incubated with horseradish peroxidase-conjugated goat anti-rabbit or goat anti-mouse IgG antibodies (Cell Signaling Technology), diluted 1:10,000, for 1 h at room temperature. The bands were visualized using SuperSignal™ West Femto (Thermo Fisher Scientific) and imaged on an enhanced chemiluminescence detection system, Amersham Biosciences equipment (A Division of GE Healthcare, Buckinghamshire, UK), according to the manufacturer’s protocols.

### 2.10. Statistical Analysis

Data are expressed as mean ± SD. Statistical analyses were performed using one-way analysis of variance (ANOVA), followed by Tukey’s multiple comparisons post hoc test. * *p*-Value < 0.05 was considered statistically significant.

## 3. Results

### 3.1. Characteristics of Olive Leaf Extract

Following lyophilization, all extracts were ultimately obtained in powder form. As the proportion of ethanol in the solvent increased, the extract color lightened from brown to greenish tones. Notably, the powder yield increased proportionally with the water content of the extraction solvent. The detailed physical characteristics, color, and yield of each extract are summarized in Table 2.

### 3.2. Inhibitory Effects of Olive Leaf Extracts on Sebum Production in SEB-1 Sebocytes

**Figure 1:**
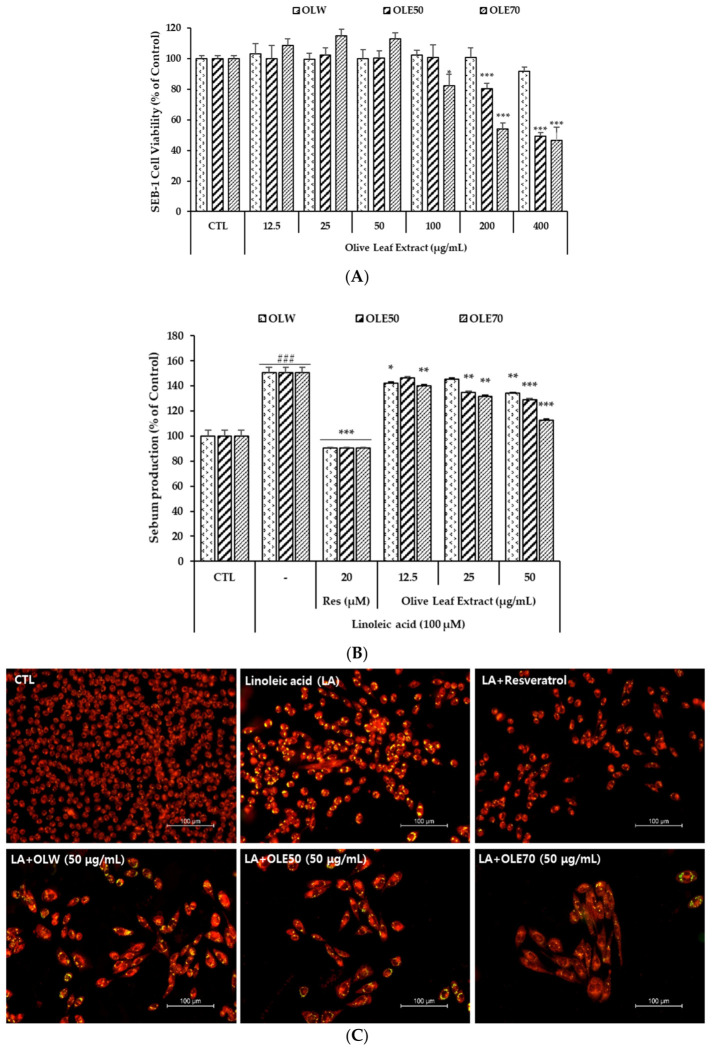
Cytotoxicity and sebum production suppression effects of olive leaf extracts in SEB-1 sebocytes. (**A**) Cell viability of SEB-1 sebocytes after 24 h exposure to the indicated concentrations (12.5–400 µg/mL). (**B**,**C**) Intracellular lipid accumulation induced by linoleic acid (100 µM) was measured using Oil Red O staining and Nile Red staining in SEB-1 sebocytes. Resveratrol (20 µM) was used as a reference lipid-lowering control to confirm assay responsiveness, based on its reported suppressive effects on lipid accumulation and lipogenesis-related signaling in human sebocyte models under lipogenic conditions [31]. Data are expressed as mean ± SD (n = 3). Statistical significance was determined by one-way ANOVA followed by post hoc test. ^###^ *p* < 0.001 vs. CTL; * *p* < 0.05; ** *p* < 0.01; *** *p* < 0.001 vs. LA-treated group.

To confirm the inhibitory effects of olive leaf extracts on sebum production in SEB-1 sebocytes, the cytotoxicity and lipid-suppressing activities of three extract types (OLW, OLE50, and OLE70) were evaluated (Figure 1A). Because the analysis whose results are reported in Figure 1 was designed for an initial screening to assess sebocyte lipogenesis/sebum production, we employed a lipogenesis-inducing condition (linoleic acid, LA) rather than *C. acnes* stimulation [37]. Accordingly, Figure 1 represents an LA-driven sebocyte lipogenesis model, not a *C. acnes*-triggered inflammatory model. Based on the viability data, non-toxic concentrations were selected for further assessment of sebum production using Oil Red O and Nile Red staining. Treatment with OLEs significantly reduced intracellular lipid accumulation in SEB-1 sebocytes without inducing cytotoxicity (Figure 1B,C). At the highest tested concentration (50 µg/mL), OLW, OLE50, and OLE70 decreased lipid accumulation by 27.62%, 36.19%, and 63.02%, respectively, compared with the linoleic acid-treated control, indicating a concentration-dependent suppression of sebum synthesis.

### 3.3. Inhibitory Effects of Olive Leaf Extracts on Pro-Inflammatory Cytokine Production in HaCaT Keratinocytes

**Figure 2:**
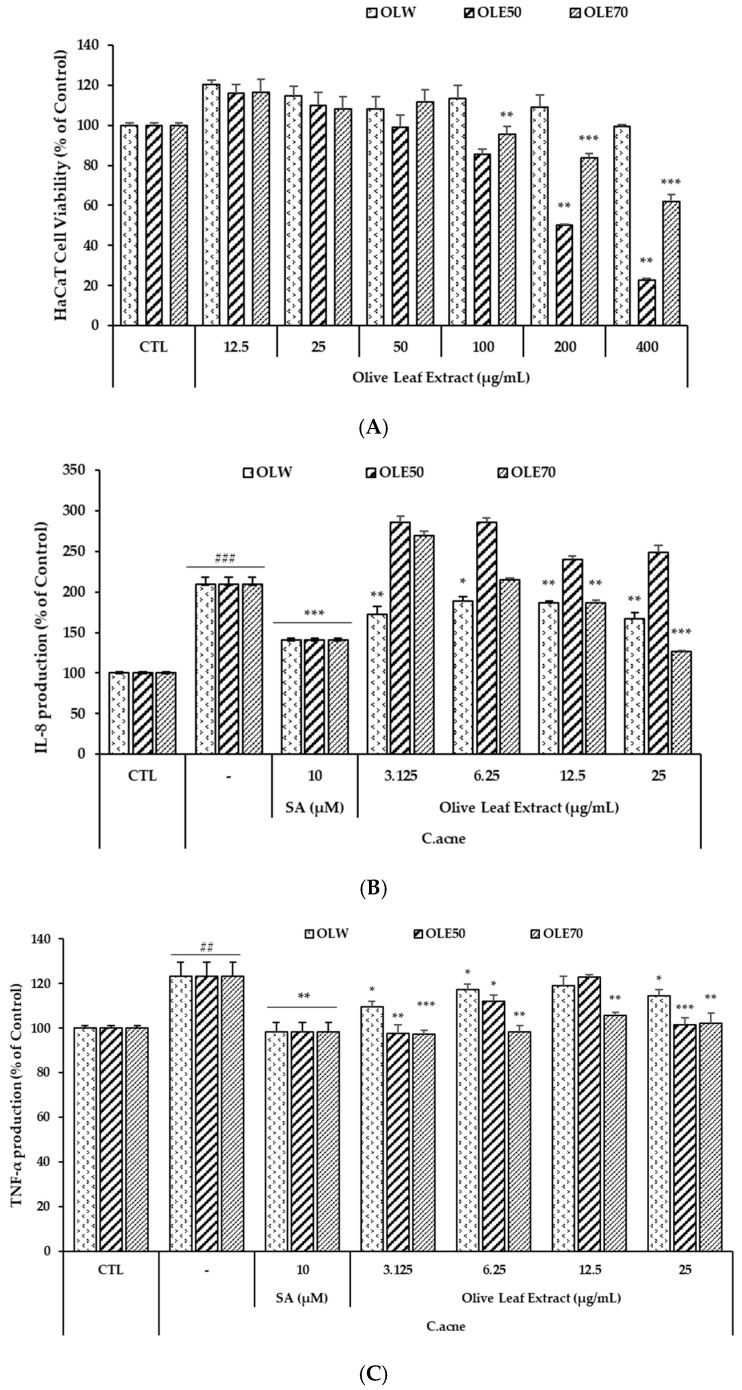

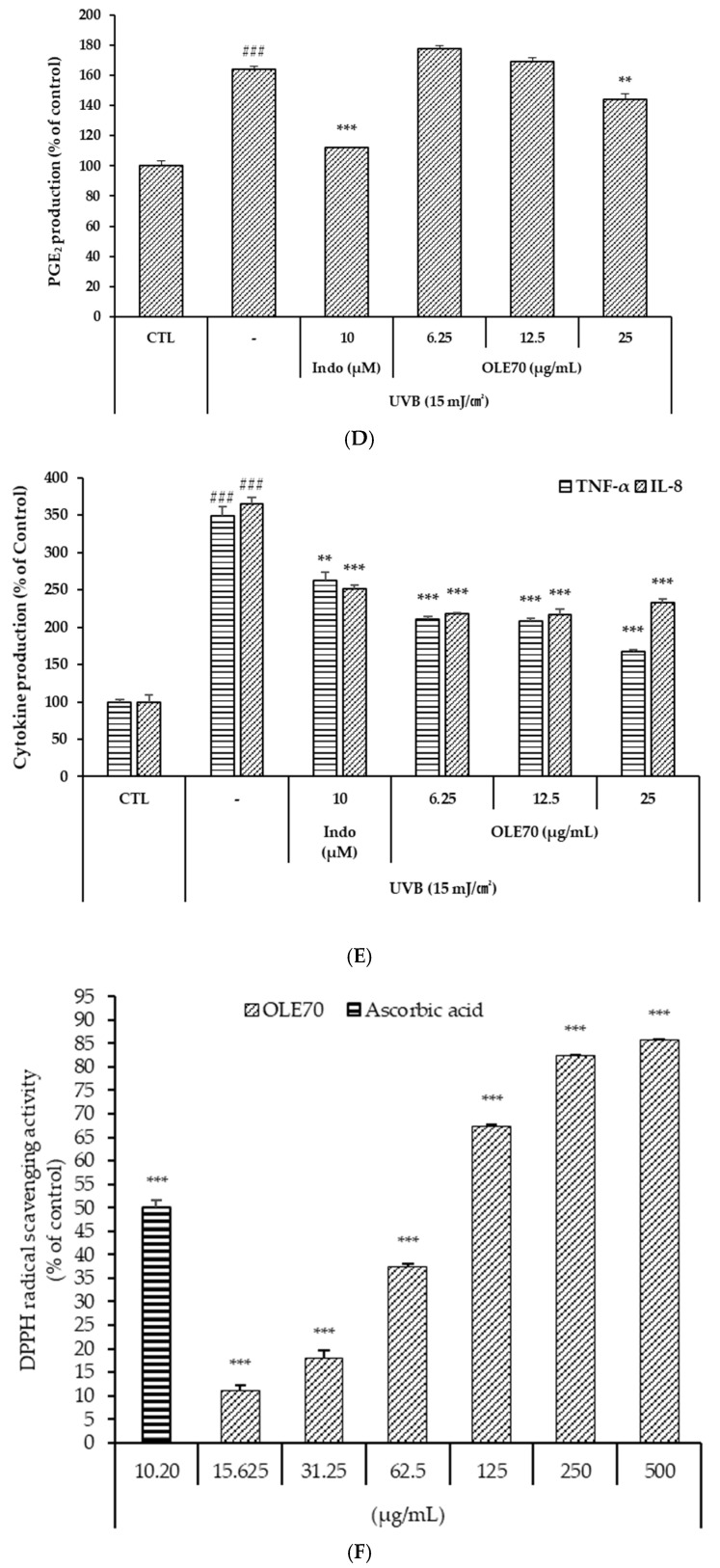
OLE alleviates *C. acnes*- and UVB-induced inflammatory cytokine production in HaCaT cells. (**A**) Cell viability of HaCaT cells treated with OLEs (12.5–400 µg/mL). (**B**,**C**) Analysis of IL-8 and TNF-α production in HaCaT cells stimulated with *C. acnes* and treated with OLEs; salicylic acid (SA) was included as an acne-related reference control for the *C. acnes*-induced inflammatory cytokine readout in HaCaT keratinocytes [32,33,34]. (**D**,**E**) Evaluation of TNF-α, IL-8, and PGE_2_ secretion in HaCaT cells exposed to UVB irradiation (15 mJ/cm^2^) and subsequently treated with OLE70; indomethacin (Indo, 10 µM) was used as a pharmacological COX-inhibitory reference control for UVB-induced COX-linked inflammatory mediator production, particularly PGE_2_ [35,36]. (**F**) Assessment of the DPPH radical-scavenging activity of OLE70 in a cell-free system. Data are expressed as mean ± SD (n = 3). Statistical significance was determined by one-way ANOVA followed by post hoc test. ^##^ *p* < 0.01, ^###^ *p* < 0.001 vs. control. (**B**,**C**) * *p* < 0.05, ** *p* < 0.01, and *** *p* < 0.001 vs. *C. acnes*-treated group. (**D**,**E**) * *p* < 0.05, ** *p* < 0.01, and *** *p* < 0.001 vs. UVB-treated group. (**F**) *** *p* < 0.001 vs. control.

To evaluate the anti-inflammatory and antioxidant activities of olive leaf extracts, HaCaT keratinocytes were used as an in vitro model. Cytotoxicity assays revealed that OLE70 exhibited cytotoxicity at 100 µg/mL, whereas OLE50 and OLW showed toxicity at 200 µg/mL (Figure 2A). Therefore, the evaluation concentrations were set below 25 µg/mL, which was the highest common non-toxic concentration. OLE treatments were applied concurrently with *C. acnes* stimulation. OLE markedly reduced *C. acnes*-induced IL-8 and TNF-α levels in a concentration-dependent manner (Figure 2B,C). In Figure 2B, at the highest tested concentration (25 µg/mL), OLW reduced *C. acnes*-induced IL-8 levels by 61.04%, while OLE70 reduced IL-8 production to levels below those of the unstimulated control, indicating a strong inhibitory effect. Similarly, as shown in Figure 2C, OLW, OLE50, and OLE70 decreased *C. acnes*-induced TNF-α levels by 35.64%, 87.75%, and 84.10%, respectively, at 25 µg/mL, demonstrating robust suppression of pro-inflammatory cytokine production by OLEs.

In addition, since OLE70 demonstrated superior efficacy among the extracts tested, it was further examined for UVB-induced inflammatory responses. Under UVB irradiation (15 mJ/cm^2^), OLE70 significantly suppressed the production of TNF-α, IL-8, and PGE_2_, exhibiting anti-inflammatory effects comparable to indomethacin (10 µM) (Figure 2D,E). Moreover, OLE70 showed potent antioxidant capacity, as indicated by its strong DPPH radical-scavenging activity (Figure 2F).

### 3.4. Dermal Elasticity-Related Effects of Olive Leaf Extracts Using Contraction Analysis of Collagen Gel Containing Fibroblasts

**Figure 3:**
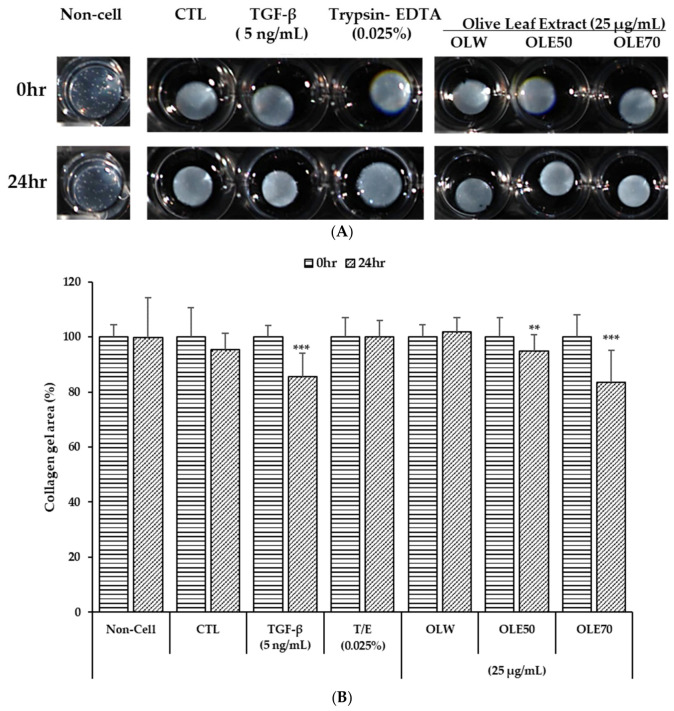
Collagen gel contraction induced by olive leaf extracts in dermal fibroblasts. (**A**) Photographs of collagen gels at 0 h and 24 h after treatment. (**B**) Quantification of gel area contraction (% of control). OLEs promoted gel contraction similar to TGF-β (5 ng/mL) treatment, suggesting enhanced fibroblast contractility. Data are expressed as mean ± SD (n = 3). Statistical significance was determined by one-way ANOVA followed by post hoc test. ** *p* < 0.01 and *** *p* < 0.001 vs. 0 h group.

To investigate the effects of olive leaf extracts (OLEs) on dermal fibroblast contractility, a collagen gel contraction assay was performed (Figure 3A,B). The collagen gels treated with trypsin–EDTA served as a negative control, as the enzymatic disruption of cell–matrix adhesion prevented fibroblasts from generating contractile forces, thereby eliminating active cell-mediated gel contraction. In contrast, TGF-β, a well-established inducer of fibroblast contraction, was used as the positive control and induced approximately 14.53% gel contraction [22]. OLE treatment markedly enhanced fibroblast-mediated gel contraction compared with the untreated control, exhibiting a contraction pattern comparable to that induced by TGF-β (Figure 3A). As shown in Figure 3B, OLW did not elicit measurable gel contraction, whereas OLE50 and OLE70 induced approximately 5.25% and 16.48% contraction, respectively. Notably, OLE70 exerted a more pronounced contractile response than the positive control (14.53%, 5 ng/mL TGF-β). These findings indicate that OLEs enhanced fibroblast-mediated collagen gel contraction under the present in vitro conditions, supporting a possible effect on dermal remodeling-related contractile responses rather than direct evidence of improved dermal firmness or anti-acne efficacy.

### 3.5. OLE70 Modulates SREBP-1/PPAR-γ and AKT/ERK Signaling in Sebocytes

**Figure 4:**
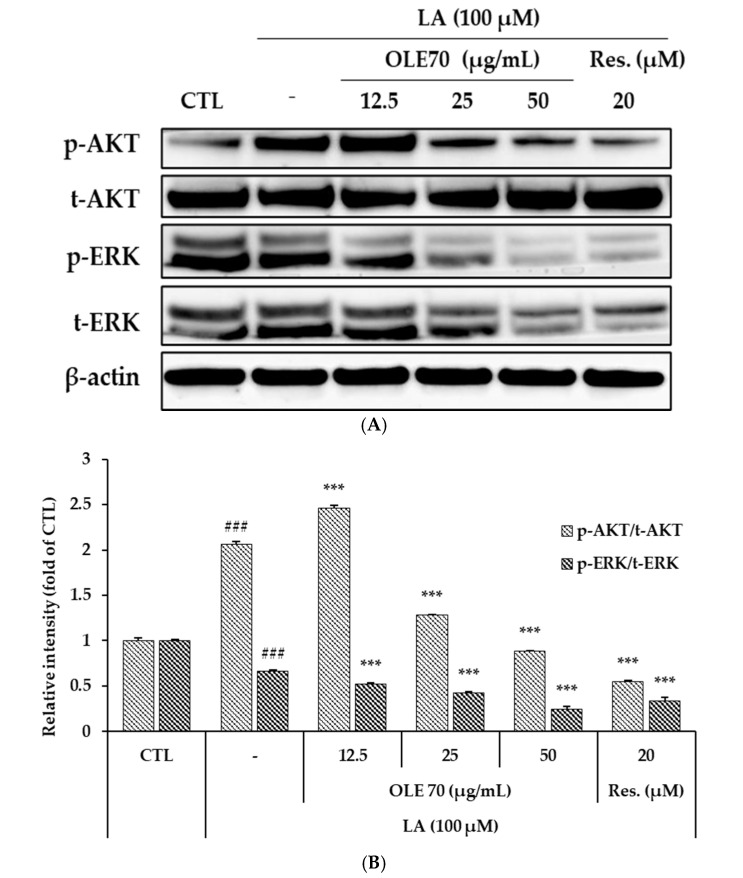

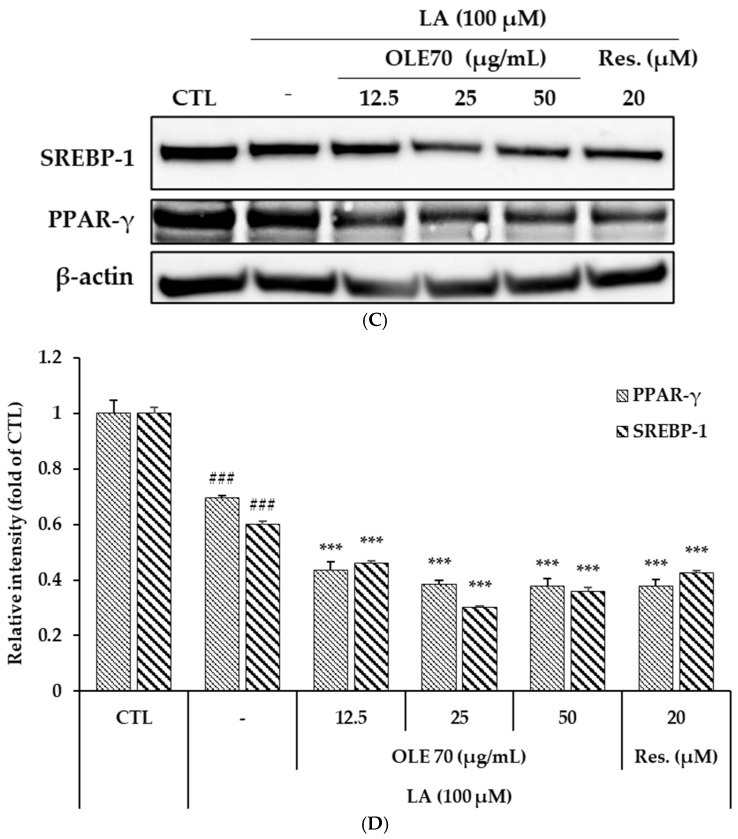
Mechanistic regulation of lipid synthesis and inflammatory signaling by OLE70 in SEB-1 sebocytes. (**A**) SEB-1 sebocytes were treated with OLE70 (12.5–50 µg/mL) for 30 min. Protein expression of phospho-AKT, total AKT, phospho-ERK, and total ERK was detected. (**B**) Quantification of phosphorylated AKT and ERK, each normalized to their respective total protein levels as shown in (**A**). (**C**) SEB-1 sebocytes were treated with OLE70 (12.5–50 µg/mL) for 24 h. Protein expression of SREBP-1 and PPAR-γ was detected. (**D**) Quantification of SREBP-1 and PPAR-γ expression levels, each normalized to β-actin, as shown in (**C**). *β*-Actin was used as loading control. Densitometric analysis showed that OLE70 downregulated SREBP-1 and PPAR-γ and reduced AKT and ERK phosphorylation, suggesting a downregulatory effect in lipid metabolism and inflammatory signaling. Data are expressed as mean ± SD (n = 3). Statistical significance was determined by one-way ANOVA followed by post hoc test. ^###^ *p* < 0.001 vs. control, and *** *p* < 0.001 vs. LA-treated group.

Next, we performed Western blot analysis of signaling molecules involved in lipid synthesis and inflammation. SEB-1 sebocytes were treated with OLE70 for either 30 min or 24 h prior to protein analysis. OLE70 treatment for 30 min dose-dependently reduced LA-induced phosphorylation of AKT and ERK, while total AKT and ERK protein levels remained unchanged (Figure 4A). The corresponding densitometric quantification of the Western blot band is presented in Figure 4B. After 24 h treatment, OLE70 markedly downregulated the expression of the lipogenic transcription factors SREBP-1 and PPAR-γ (Figure 4C), and the quantitative analysis of the expression levels of these proteins is shown in Figure 4D. Collectively, these results indicate that OLE70 suppresses lipid metabolic and inflammatory signaling pathways by attenuating AKT/ERK activation and reducing the expression of key regulators of lipogenesis.

### 3.6. Antioxidant and Anti-Sebum Effects of Oleuropein

Oleuropein is a bioactive phenolic compound derived from olive (*Olea europaea* L.) leaves and is well known for its broad pharmacological activities. In a 2,4-dinitrochlorobenzene (DNCB)-induced atopic dermatitis mouse model, Huang et al. demonstrated that oleuropein markedly attenuates cutaneous inflammation and regulates immune responses. Consistent with this, oleuropein has been reported to exert anti-inflammatory, antioxidant, anti-obesity, anti-diabetic, anti-cancer, and hepatoprotective properties, highlighting its potential relevance to metabolic and inflammatory conditions. In dermatological research, oleuropein has additionally been shown to promote wound healing, prevent apoptosis in irradiated keratinocytes, and improve skin elasticity [38]. Given these diverse biological activities, we sought to examine whether the functional effects observed with OLEs could be recapitulated by oleuropein itself; therefore, we conducted a series of assays to evaluate oleuropein content and its biological efficacy independently. These pleiotropic skin-related effects suggest that oleuropein may beneficially influence sebum regulation and oxidative or inflammatory pathways in sebocytes and keratinocytes.

**Figure 5:**
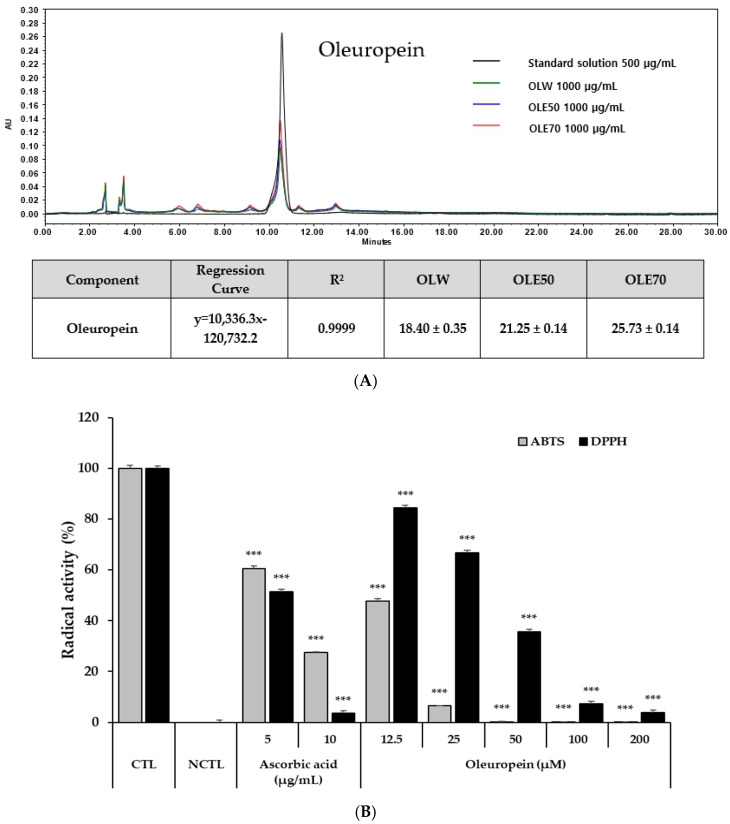

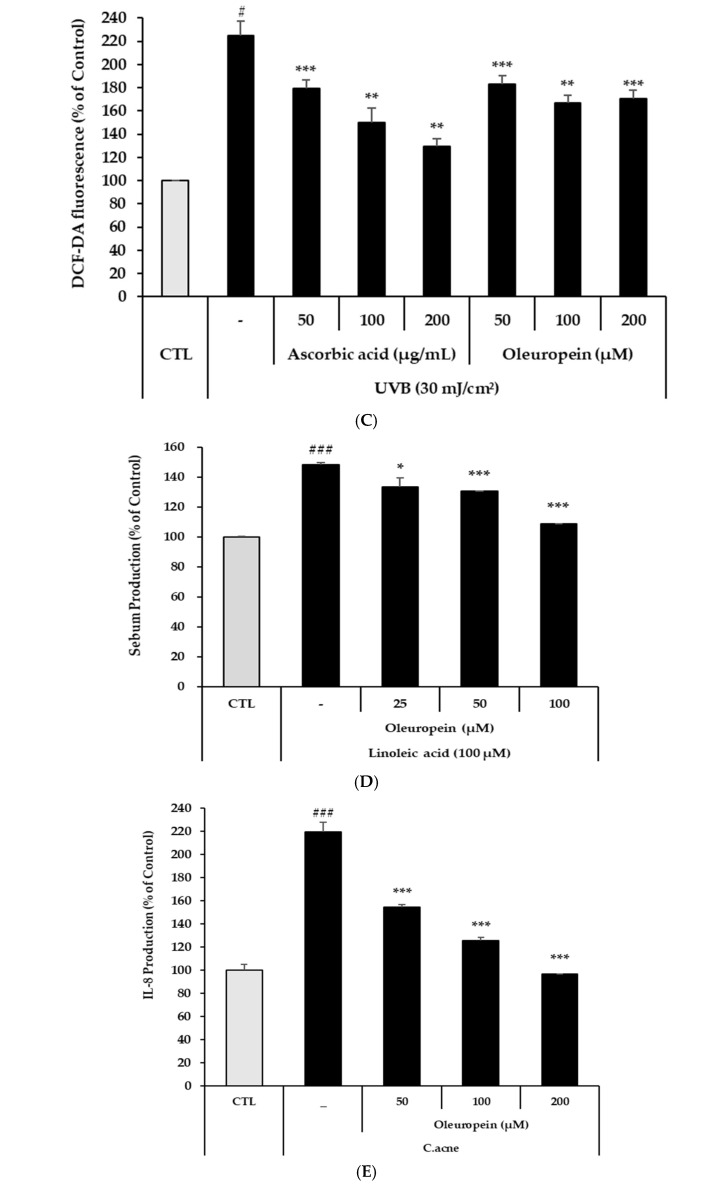

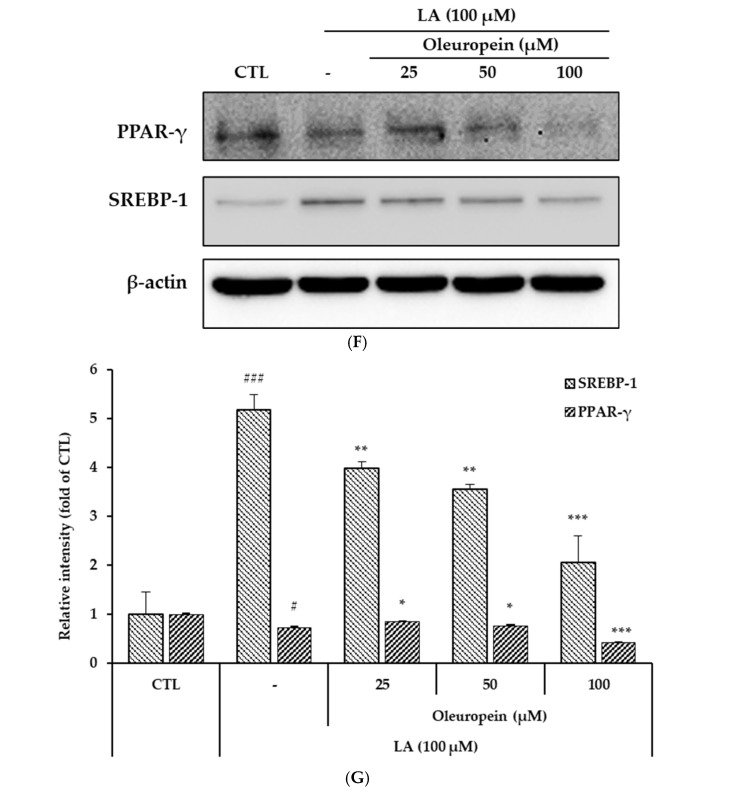
Oleuropein exhibits strong antioxidant activity and suppresses sebum production. (**A**) Representative HPLC chromatograms of oleuropein in the standard solution and three extracts, with quantification of oleuropein content in each extract. (**B**) DPPH and ABTS radical-scavenging activities of oleuropein compared with ascorbic acid. (**C**) ROS levels in UVB-irradiated HaCaT cells measured by DCF-DA fluorescence were reduced by oleuropein. (**D**) Intracellular lipid accumulation induced by linoleic acid (100 µM) in SEB-1 sebocytes was measured using Oil Red O staining. (**E**) Oleuropein significantly reduced *C. acnes*-induced IL-8 production in HaCaT cells. (**F**) Western blot analysis showing SREBP-1 and PPAR-γ suppression in SEB-1 cells treated with oleuropein (25–100 µM) for 24 h. (**G**) Quantification of PPAR-γ and SREBP-1 expression levels, each normalized to β-actin, as shown in (**F**). *β*-Actin was used as loading control. Data are presented as mean ± SD (n = 3). Statistical significance was determined by one-way ANOVA followed by post hoc test. (**B**) *** *p* < 0.001 vs. control. (**C**) ^#^ *p* < 0.05 vs. control; ** *p* < 0.01, and *** *p* < 0.001 vs. UVB-treated group. (**D**) ^###^ *p* < 0.001 vs. control; * *p* < 0.05, and *** *p* < 0.001 vs. LA-treated group. (**E**) ^###^ *p* < 0.001 vs. control; and *** *p* < 0.001 vs. *C. acnes*-treated group. (**G**) ^#^ *p* < 0.05, and ^###^ *p* < 0.001 vs. control; * *p* < 0.05, ** *p* < 0.01, and *** *p* < 0.001 vs. LA-treated group.

The oleuropein reference standard was eluted at a retention time of 10.57 min under the established chromatographic conditions. The standard calibration curve exhibited excellent linearity across the tested range of 62.5–1000 μg/mL, with an R^2^ value of 0.9999. The LOD and LOQ values were determined to be 11.89 μg/mL and 36.02 μg/mL, respectively. Inter-day precision, assessed by repeated analysis of a 250 μg/mL oleuropein standard solution over three consecutive days, showed an RSD of less than 2%, demonstrating acceptable repeatability. Quantitative analysis revealed that oleuropein accounted for 18.40 ± 0.35%, 21.25 ± 0.14%, and 25.73 ± 0.14% (*w*/*w*) of the OLW, OLE50, and OLE70 dry extracts, respectively. Among the tested extracts, OLE70 contained the highest oleuropein content, indicating that 70% ethanolic extraction was the most efficient condition for oleuropein enrichment (Figure 5A).

Prior to functional evaluation, oleuropein was assessed for cytotoxicity in SEB-1 sebocytes and HaCaT keratinocytes, and no significant cytotoxicity was observed at the concentrations used in this study (Figure S1). Oleuropein exhibited strong antioxidant capacity in ABTS, DPPH, and DCF-DA assays, showing robust radical-scavenging and intracellular ROS-reducing activities comparable to or exceeding those of OLEs (Figure 5B,C).

In SEB-1 sebocytes, oleuropein significantly reduced linoleic acid-induced lipid accumulation and suppressed *C. acnes*-stimulated IL-8 secretion (Figure 5D,E). Mechanistically, oleuropein downregulated SREBP-1 and PPAR-γ expression in SEB-1 cells (Figure 5F), indicating that oleuropein suppresses sebum production and modulates inflammatory responses by targeting key lipogenic and transcriptional regulators.

## 4. Discussion

Olive leaf extract (OLE) and its major phenolic compound, oleuropein, were found to exert dual modulatory effects on lipid synthesis and inflammatory signaling in skin cells, suggesting their potential as bioactive ingredients for acne-prone and oily skin formulations. The suppression of SREBP-1 and PPAR-γ observed in this study suggests that OLE and oleuropein may regulate sebaceous lipid metabolism by attenuating excessive lipid synthesis while contributing to lipid homeostasis [13,14]. Downregulation of PPAR-γ, LXRα/β, and SREBP-1 expression has been reported to suppress sebaceous lipogenesis and differentiation in sebocytes [39], which is consistent with our observation that OLE70 appears to modulate these key transcriptional regulators. These findings are in line with previous reports suggesting that modulation of the SREBP-1/PPAR-γ axis can attenuate sebocyte hyperactivity and inflammation [13,14]. Furthermore, our observation that OLE70 effectively downregulated SREBP-1 and PPAR-γ in SEB-1 sebocytes provides supportive rather than definitive evidence that OLE may exert sebum-suppressive activity through transcriptional modulation, complementing the findings obtained by Kimura et al. [16], who reported similar lipid-regulatory trends in UVB-challenged skin models. Notably, human clinical-cell evidence indicates that pharmacological activation of PPAR subtypes can be associated with increased sebaceous lipogenesis, emphasizing that selective or temporal modulation—rather than broad activation—may be critical when translating PPAR-γ biology to acne management [14]. Consistent with this concept, therapeutic suppression of SREBP-1 has been shown to down-tune sebocyte lipogenesis; for example, salicylic acid has been reported to reduce sebogenesis by inhibiting the AMPK/SREBP-1 axis in SEB-1 sebocytes [40]. In the present study, linoleic acid was used as a controlled lipogenic stimulus to interrogate canonical sebocyte regulators, whereas pathogen-linked inflammatory relevance was addressed separately in keratinocytes under *C. acnes* stimulation [37,40]. This interpretation is also supported by evidence that TNF-α can directly enhance sebocyte lipogenesis through JNK and PI3K/Akt signaling, highlighting mechanistic crosstalk between inflammatory and lipogenic pathways in sebocytes [41].

Although oleuropein was used as the principal quantitative marker compound in this study based on previous reports identifying it as a predominant phenolic/secoiridoid constituent of olive leaves and a well-characterized bioactive compound, OLE should be interpreted as a chemically complex phytochemical mixture rather than as a single-compound preparation. Other olive leaf-derived phenolics, including hydroxytyrosol, tyrosol, verbascoside, luteolin glycosides, and apigenin glycosides, have also been identified in olive leaves and may contribute to the observed biological activities through additive or synergistic mechanisms [42,43,44]. Therefore, the present findings do not establish oleuropein as the sole active constituent responsible for the effects of OLE. Further comprehensive LC-MS-based phenolic profiling and activity-guided fractionation will be required to clarify the relative contribution of individual constituents to the sebum-regulatory, antioxidant, and anti-inflammatory effects of OLE.

Although OLE has been reported in some studies to exhibit antimicrobial activity against *Cutibacterium acnes* and *Staphylococcus epidermidis* [45], such activity was not detectable under the present experimental conditions (Figure S2, Table S1, Supplementary Data). This discrepancy may be attributable to differences in extraction composition, phenolic profile, or assay sensitivity. Comparable variability has been noted by Borjan et al. [18], who reported that the antimicrobial potency of olive phenolics varies substantially depending on solvent polarity and extraction conditions. Accordingly, while certain extracts may demonstrate bactericidal or bacteriostatic effects, our findings suggest that the functional efficacy of OLE70 primarily resides in anti-inflammatory, sebum-regulatory, and antioxidant properties rather than direct antibacterial activity. This contrasts with previous work by Sudjana et al. [46] showing measurable MIC values against Gram-positive species, highlighting how extraction method and polyphenol spectrum critically determine biological outcomes.

Importantly, the present study was not designed to demonstrate direct clinical anti-acne efficacy. The linoleic acid-induced SEB-1 sebocyte model was used to evaluate sebocyte lipogenesis, whereas the *C. acnes*-stimulated HaCaT keratinocyte model was used to assess inflammatory mediator production under a pathogen-relevant inflammatory condition. Therefore, the observed suppression of lipid accumulation and the reduction in *C. acnes*-induced inflammatory mediators should be interpreted as modulation of acne-associated biological factors rather than as direct evidence of acne improvement. Nevertheless, because excessive sebum production and *C. acnes*-associated inflammation are key features of oily and acne-prone skin, these findings suggest that OLE70 may have potential relevance for sebum control and inflammatory management in this skin context. Further studies using more acne-relevant experimental systems, such as androgen- or *C. acnes*-stimulated sebocytes, sebocyte–keratinocyte co-culture models, and clinical studies in subjects with oily or acne-prone skin, will be required to determine whether these in vitro effects translate into improvement of acne-related skin conditions.

In addition, the positive controls used in the present study should be interpreted as assay- or endpoint-oriented reference controls rather than as compounds with mechanisms identical to those of OLE. Resveratrol was included as a reference lipid-lowering control to confirm the responsiveness of the linoleic acid-induced lipid-accumulation assay [28,29,30,31], whereas salicylic acid and indomethacin were used to validate inflammatory readouts associated with *C. acnes*-stimulated cytokine production and UVB-induced COX/PGE_2_-linked responses, respectively [32,33,34,35,36]. Future studies including additional anti-inflammatory reference controls, such as nicotinamide, may further strengthen the mechanistic interpretation of OLE in C. acnes-associated inflammatory models.

To clarify the physiological relevance of the UV irradiation condition, UVB exposure was not intended to model UV as a primary etiologic trigger of acne; rather, it was used as a standardized external stressor to robustly engage the COX-2–PGE_2_ inflammatory axis, which is mechanistically implicated in pilosebaceous inflammation and sebaceous gland activity. In human sebocytes (SZ95), UVB and oxidative stress have been shown to activate a PPAR-γ dependent program leading to COX-2 induction and increased PGE_2_ production [47]. In parallel, UVB has also been reported to enhance sebaceous lipogenesis/sebum production in sebaceous models, further supporting its use as a standardized stressor relevant to acne-associated inflammatory and sebaceous responses [48]. Likewise, UVB induces COX-2 expression and PGE_2_ generation in primary human keratinocytes, and this response is attenuated by pharmacological inhibition of PPAR-γ or COX-2, supporting UVB as a reproducible stimulus for COX-2–PGE_2_-mediated inflammatory signaling in epidermal cells [49]. UVB is also known to induce pro-inflammatory chemokines such as IL-8 and MCP-1 in keratinocytes, further supporting its use as a standardized epidermal inflammatory stressor in the present study [50]. Beyond UV, sebocytes can also upregulate COX-2/PGE_2_ and release pro-inflammatory mediators (e.g., IL-8) in response to non-microbial inflammatory cues such as platelet-activating factor receptor activation, indicating that COX-2–PGE_2_ induction represents a broader stress-responsive inflammatory program within the pilosebaceous unit [51]. Clinically, increased COX-2 expression has been reported in sebaceous glands from acne-involved skin [47,51]. Furthermore, in vivo evidence links this pathway to sebaceous output: constitutive COX-2 expression in mouse skin leads to sebaceous gland hyperplasia and increased sebum accumulation at the skin surface [52]. Collectively, these data support the use of UVB as a controlled experimental trigger to interrogate acne-relevant COX-2–PGE_2_ signaling and its modulation by test materials [47,49,51,52]. Accordingly, suppression of UVB-induced inflammatory outputs in HaCaT keratinocytes in the present study should be interpreted as evidence of epidermal anti-inflammatory activity relevant to acne-linked inflammatory signaling, rather than as evidence that ultraviolet exposure is a primary etiologic trigger of acne [49].

A notable observation of this study is the inhibition of TNF-α, IL-8, and PGE_2_ production in UVB-irradiated HaCaT keratinocytes by OLE70. This result suggests that OLE70 may attenuate cytokine-mediated inflammatory responses in epidermal cells under oxidative stress conditions. These observations are consistent with previous studies reporting that olive leaf phenolics, including oleuropein, can exert photoprotective and anti-inflammatory effects by suppressing UVB-induced COX-2 and MMP expression [53]. In contrast to studies focusing primarily on photoprotection, the present results indicate that OLE70 may concurrently influence cytokine secretion and lipid oxidation, suggesting a dual regulatory tendency relevant to oily and acne-prone skin. Similar patterns have been described by Aparicio-Soto et al. [54], who reported inhibition of NF-κB signaling in keratinocytes by olive oil-derived polyphenols.

Acne-associated inflammation is mediated by a complex network of pro-inflammatory cytokines and chemokines produced by keratinocytes and sebocytes. Among these, IL-8 and TNF-α are widely recognized as key inflammatory mediators in acne pathogenesis, playing critical roles in neutrophil recruitment and amplification of inflammatory responses within the pilosebaceous unit [21,55]. For this reason, the present study primarily focused on these representative cytokines. In our experiments, oleuropein significantly suppressed IL-8 production, whereas no inhibitory effect was observed on TNF-α under the same experimental conditions. This selective response suggests that oleuropein may exert a cytokine-specific anti-inflammatory effect rather than broad-spectrum cytokine suppression. In contrast, olive leaf extracts, which contain multiple bioactive constituents, may modulate inflammatory responses through additive or synergistic interactions among their components. Such differences between complex natural extracts and single isolated compounds have been reported previously and may explain the distinct cytokine modulation profiles observed in this study [21,55]. Although other pro-inflammatory cytokines, such as IL-6, are also involved in acne-related inflammation, the current findings suggest that oleuropein preferentially targets IL-8-mediated inflammatory pathways. However, further studies will be required to comprehensively evaluate the effects of oleuropein and olive leaf extracts on a broader cytokine panel and to elucidate their distinct anti-inflammatory mechanisms. Consistent with the microbiome-linked inflammatory component of acne, OLE70 significantly attenuated inflammatory mediator outputs in keratinocytes under *C. acnes* stimulation (Figure 2B,C), supporting anti-inflammatory relevance in a pathogen-triggered context [56]. Thus, pathogen-linked inflammation and sebaceous lipogenesis were addressed as complementary axes in the present study.

This axis-aligned allocation of endpoints across different skin cell systems is consistent with published anti-acne and skin-biology studies [57,58]. For example, Kaempferia parviflora extract was evaluated using a multi-component framework combining keratinocyte inflammatory readouts with sebocyte lipogenesis/PPAR-γ modulation. Likewise, hemp seed hexane extracts were studied using Propionibacterium acnes-stimulated HaCaT keratinocytes for inflammatory outputs together with sebocyte lipogenesis assays, and platycodin D was reported to modulate sebocyte lipogenesis, inflammatory responses, and collagen-related remodeling in separate but biologically relevant systems. These precedents support the scientific acceptability of distributing acne-relevant functional axes across complementary cell models according to their primary biological relevance [59,60,61].

Our findings are generally consistent with accumulating evidence suggesting that oleuropein and related olive phenolics attenuate oxidative and inflammatory signaling in human skin cells and fibroblasts [20,62]. In addition to sebocyte- and keratinocyte-mediated mechanisms, acne pathophysiology is increasingly recognized to involve structural alterations within the dermal compartment. Chronic inflammatory acne is associated with extracellular matrix degradation, reduced collagen density, and impaired dermal remodeling, which contribute not only to acne scarring but also to premature skin aging. Such collagen loss and decreased dermal integrity may exacerbate follicular deformation and prolong inflammatory lesion persistence [63,64].

The fibroblast collagen gel contraction assay was included only as a dermal remodeling/contractility endpoint relevant to post-inflammatory structural support, rather than as a direct model of acne pathogenesis. This interpretation is supported by published studies showing that inflammatory acne lesions are accompanied by collagen degradation and extracellular matrix remodeling in vivo [23], while persistent acne scarring is associated with reduced dermal thickness and loss of pilosebaceous units [25]. In addition, *C. acnes* has been shown to induce TNF-α-dependent proMMP-2 expression in human dermal fibroblasts, further supporting a mechanistic connection between acne-associated inflammation and dermal matrix remodeling [26]. Collagen gel contraction has been widely used as an in vitro wound-healing/remodeling model, and TGF-β is a recognized positive inducer of fibroblast-mediated collagen gel contraction [24]. In this context, the enhanced contraction observed with OLE70 is best interpreted as supportive evidence for fibroblast-mediated dermal remodeling/contractility and structural support, rather than as direct evidence of anti-acne efficacy. Dermal fibroblasts and myofibroblasts play key roles in matrix remodeling, mechanotransduction, and contractile activity during tissue repair [65].

Another notable outcome is that OLE70 treatment was associated with enhanced collagen gel contraction in a fibroblast–collagen 3D model, suggesting a potential enhancement of dermal remodeling capacity. This observation is in agreement with previous reports indicating that olive-derived phenolic or PPAR-related complexes may promote collagen synthesis and ECM reorganization in dermal models [25,27]. Accordingly, SEB-1 sebocytes, HaCaT keratinocytes, and dermal fibroblasts were used as complementary cell models to capture distinct skin-relevant axes, while mechanistic interrogation was prioritized in sebocytes as the primary lipogenesis-related module.

Beyond these effects, OLE exhibited antioxidant activity, which may contribute to protection against oxidative stress and UVB-induced cellular damage, consistent with previous observations reported by Kimura et al. [16]. Collectively, the combination of antioxidative, anti-inflammatory, and remodeling-associated activities suggests that OLE70 may function as a multifunctional natural ingredient for sebum control, skin soothing, and structural support. However, these findings should be interpreted as exploratory, and further mechanistic and translational studies are warranted to substantiate these effects [14,16].

In summary, the present study provides integrative and exploratory insights that bridge sebocyte lipid regulation with keratinocyte and fibroblast responses, while maintaining consistency with prior reports on olive-derived phenolics [20,62]. These results suggest a potential role of OLE70 in supporting skin homeostasis and indicate its possible relevance for sebum-regulatory, inflammatory, and photoaging-related dermocosmetic applications. Importantly, this study is framed in an axis-aligned manner, combining the mechanistic mapping of sebocyte lipogenesis with pathogen-triggered functional readouts in keratinocytes while avoiding overextension of pathway-level claims across all cell types.

Future studies may extend the present axis-aligned framework in two directions. First, the sebocyte mechanism module can be strengthened by directly interrogating the SREBP-1/PPAR-γ axis using genetic or decoy-based approaches; notably, SREBP-1/PPAR-γ chimeric decoy oligodeoxynucleotides have shown pronounced reductions in lipogenesis and inflammatory signaling in IGF-1-stimulated sebocytes and in *C. acnes*-induced acne-like lesions in vivo [13]. Second, to more directly integrate microbiome–lipogenesis crosstalk, it will be informative to assess *C. acnes*-responsive signaling and downstream lipogenesis–inflammation coupling in sebocytes under pathogen-challenged conditions. In keratinocytes, deeper examination of NF-κB/MAPK and COX-2/PGE_2_-linked signaling under UVB and microbial triggers may further clarify whether OLE70’s cytokine-suppressive actions operate through canonical stress-response cascades, consistent with reports that olive-derived phenolic complexes modulate NF-κB and redox-related inflammatory pathways [66].

Finally, the potential microbiome-modulating effects of OLE70 remain unexplored. Botanical extracts have recently been shown to alter the ecological balance between commensal Staphylococcus epidermidis and pathogenic skin bacteria at sub-MIC concentrations, enhancing SCFA production and suppressing pathogen biofilm formation without direct bactericidal activity [67]. Similar approaches could determine whether OLE70 exerts ecological rather than purely antimicrobial benefits that contribute to the management of sebum- and inflammation-associated features of oily or acne-prone skin. Collectively, these future directions may refine the mechanistic understanding of OLE70 and expand its relevance as a multifunctional dermocosmetic ingredient in acne, inflammation-related disorders, and photoaging.

## 5. Conclusions

Taken together, our results suggest that olive leaf extract (OLE) and its major phenolic constituent, oleuropein, may exert multi-targeted modulatory effects in sebaceous and epidermal cells through coordinated regulation of SREBP-1, PPAR-γ, and AKT/ERK signaling pathways. OLE70 appeared to be the most effective extract in reducing sebum accumulation in SEB-1 sebocytes, attenuating *C. acnes*- and UVB-induced inflammatory cytokine production, and enhancing antioxidant capacity without detectable cytotoxicity. In dermal fibroblast models, OLE treatment was associated with increased collagen gel contraction, suggesting a potential benefit for dermal remodeling and skin firmness. Oleuropein itself exhibited antioxidant and sebum-suppressive tendencies, supporting its contribution to the observed bioactivity of OLE. Collectively, these findings indicate that OLE and oleuropein exert complementary bioactivities across multiple skin-relevant axes. However, the present study is exploratory in nature, and further mechanistic, translational, and formulation-level studies are needed to clarify their relevance in applied skin-care contexts.

## Figures and Tables

**Table 1:** Mobile-phase gradient conditions for the HPLC determination of oleuropein.

Time (Min)	Solvent A (%)	Solvent B (%)
0	80	20
15	60	40
25	20	80
30	80	20

**Table 2:** Summary of extraction results.

	Extraction Solvent	Color	Yield (%)
OLW	Water	Dark Brown	40.72
OLE50	50% Ethanol	Brown	38.25
OLE70	70% Ethanol	Greenish brown	24.64

## Data Availability

The original contributions presented in this study are included in the article. Further inquiries can be directed to the corresponding author.

## Supplementary Materials

The following supporting information can be downloaded at: https://www.mdpi.com/article/10.3390/cimb48060549/s1.

## Abbreviations

The following abbreviations are used in this manuscript:

OLEs	Olive leaf extracts
OLW	Olive leaf aqueous extract
OLE50	Olive leaf 50% (*w*/*w*) ethanolic extract
OLE70	Olive leaf 70% (*w*/*w*) ethanolic extract
SREBP-1	Sterol regulatory element binding protein 1
PPAR-γ	Peroxisome proliferator-activated receptor γ
SEB-1	Human sebocytes
*C. acnes*	*Cutibacterium acnes*
