# Supplementary material for: Olive Leaf Extract Suppresses Sebogenesis and Inflammation via AKT/ERK and SREBP-1/PPAR-γ Signaling in Human Sebocytes
Source: Curr Issues Mol Biol. 2026 May 23;48(6):549. doi: 10.3390/cimb48060549 (PMC13298046; doi:10.3390/cimb48060549)
Supplement: Supplementary file 1 [file cimb-48-00549-s001.zip › cimb-4283375-supplementary.pdf]

Supplementary Data

Figure S1. Cytotoxicity of Oleuropein in SEB-1 and HaCaT cells

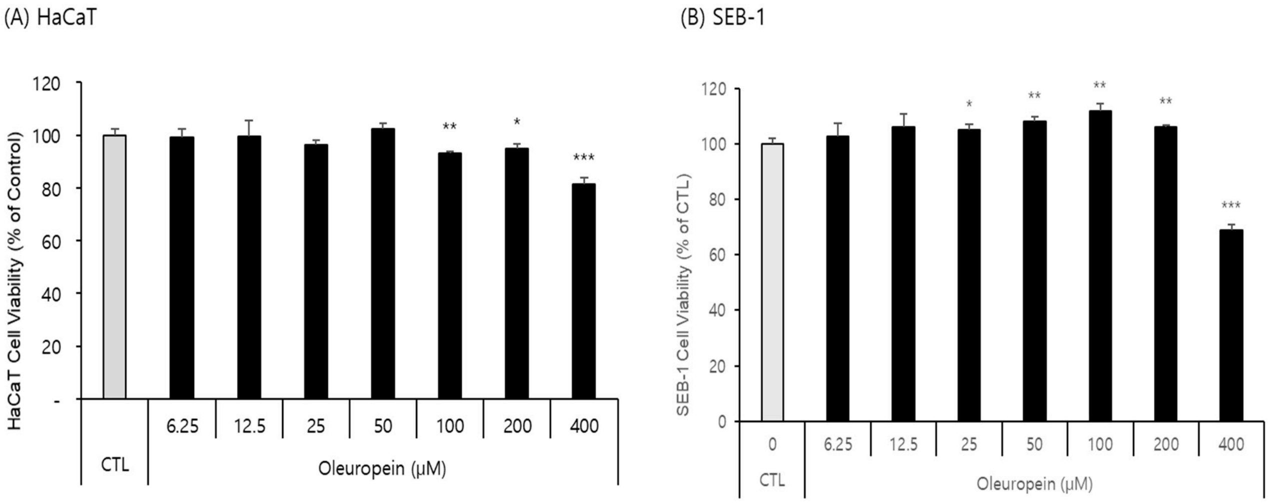

Figure S1. Cell viability of HaCaT and SEB-1 cells treated with Oleuropein. Data are expressed as mean ± SD (n = 3). *, $p < 0.05$; **, $p < 0.01$; ***, $p < 0.001$ vs. control.

Figure S2. Paper disc diffusion test

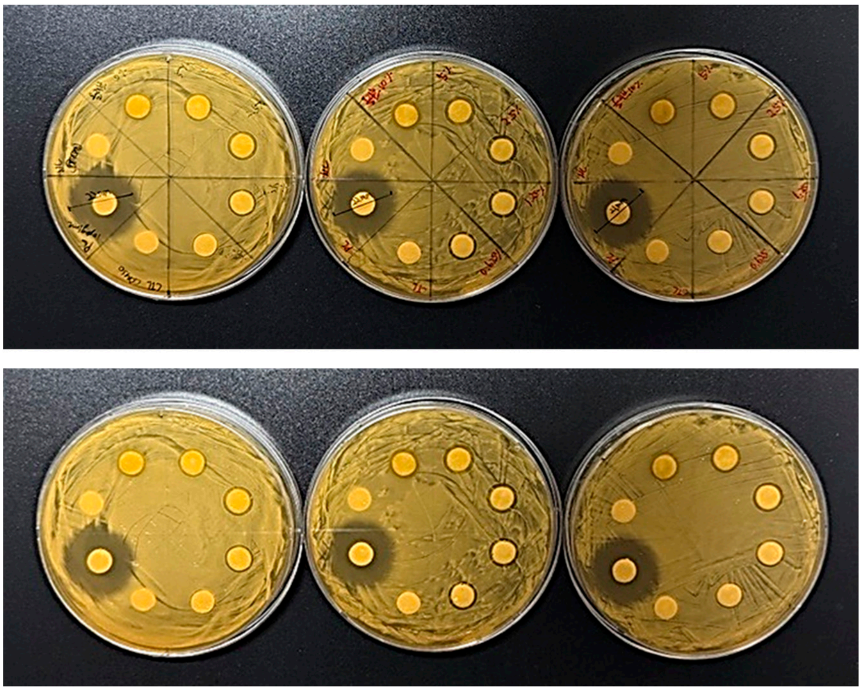

Figure S2. Inhibition zone measurements following the paper disc diffusion assay. The OLE70 produced no detectable inhibition zones against C. acnes. The vehicle control (DMSO) likewise showed no growth inhibition. In contrast, the positive control, erythromycin (10 μg/mL), yielded a mean inhibition diameter of 21.67 mm, confirming assay validity.

Table S1. Inhibition zone measurements following the paper disc diffusion assay

Strain	Concentration	Sample	Diameter of Inhibition zone (mm)
C. acnes KCTC3314	Untreated group	control	-
	10 μg/mL	erythromycin	21.67 ± 0.47
	≥99.00%	DMSO	-
	6.25 mg/mL	OLE70	-
	12.5 mg/mL		-
	25 mg/mL		-
	50 mg/mL		-
	100 mg/mL		-
